# Surface-mediated high antioxidant and anti-inflammatory effects of astaxanthin-loaded ultrathin graphene oxide film that inhibits the overproduction of intracellular reactive oxygen species

**DOI:** 10.1186/s40824-022-00276-4

**Published:** 2022-07-06

**Authors:** Seon Yeong Chae, Rowoon Park, Suck Won Hong

**Affiliations:** 1 Engineering Research Center for Color-Modulated Extra-Sensory Perception Technology, Pusan National University, Busan, 46241 Republic of Korea; 2 Department of Cogno-Mechatronics Engineering, Department of Optics and Mechatronics Engineering, College of Nanoscience and Nanotechnology, Pusan National University, Busan, 46241 Republic of Korea

**Keywords:** Astaxanthin, Graphene oxide, Antioxidant, Anti-inflammation, Wound healing

## Abstract

**Background:**

Astaxanthin (AST) is known as a powerful antioxidant that affects the removal of active oxygen and inhibits the production of lipid peroxide caused by ultraviolet light. However, it is easily decomposed by heat or light during production and storage because of the unsaturated compound nature with a structural double bond. The activity of AST can be reduced and lose its antioxidant capability. Graphene oxide (GO) is an ultrathin nanomaterial produced by oxidizing layered graphite. The chemical combination of AST with GO can improve the dispersion properties to maintain structural stability and antioxidant activity because of the tightly bonded functionalized GO surface.

**Methods:**

Layered GO films were used as nanocarriers for the AST molecule, which was produced via flow-enabled self-assembly and subsequent controlled solution deposition of RGD peptide and AST molecules. Synthesis of the GO-AST complex was also carried out for the optimized concentration. The characterization of prepared materials was analyzed through transmission electron microscopy (TEM), scanning electron microscope (SEM), Fourier-transform infrared spectroscopy (FT-IR), atomic force microscope (AFM), and Raman spectroscopy. Antioxidant activity was tested by 2,2′-azino-bis(3-ethylbenzothiazoline-6-sulfonic acid) (ABTS) and 2.2-diphenyl-1-picrylhydrazyl (DPPH) assays. The antibacterial effect and antioxidant effects were monitored for the ultrathin GO/RGD/AST Film. Further, reactive oxygen species (ROS) assay was used to evaluate the anti-inflammatory effects on L-929 fibroblasts.

**Results:**

Cotreatment of GO-AST solution demonstrated a high antioxidant combined effect with a high ABTS and DPPH radicals scavenging activity. The GO/RGD/AST film was produced by the self-assembly process exhibited excellent antibacterial effects based on physicochemical damage against *E. coli* and *S. aureus*. In addition, the GO/RGD/AST film inhibited H_2_O_2_-induced intracellular ROS, suppressed the toxicity of lipopolysaccharide (LPS)-induced cells, and restored it, thereby exhibiting strong antioxidant and anti-inflammatory effects.

**Conclusion:**

As GO nanocarrier-assisted AST exerted promising antioxidant and antibacterial reactions, presented a new concept to expand basic research into the field of tissue engineering.

**Supplementary Information:**

The online version contains supplementary material available at 10.1186/s40824-022-00276-4.

## Introduction

Astaxanthin (AST) is a type of xanthophyll carotenoid and is a secondary metabolite that is naturally synthesized by bacteria, microalgae, yeast, and the like [1, 2]. It is protected against photooxidation by ultraviolet (UV) light because it is a powerful antioxidant, has anti-inflammatory and anti-cancer effects, and is significantly involved in diabetes and aging prevention and immune response [2–6]. Therefore, AST has been actively used in skin biology and dermatology because of the potential benefits exerted on skin homeostasis [1]. Particularly, AST is known to have higher physiological activity than other carotenoids such as zeaxanthin and lutein, and higher antioxidant activity than vitamin E and β-carotene [1, 2, 4, 5]. These benefits of AST are because of its unique chemical properties and structural differences [7, 8]. AST is fat-soluble but has both hydrophilic and lipophilic properties, and has a keto group and a hydroxyl group at each end of the molecule [9, 10]. The unique chemical properties and structure of AST with long conjugated double bonds have shown strong antioxidant efficacy. In specific, it can be connected from the inside of the cell membrane to the outside, so it expresses better activity than other antioxidants. The polyene chain of AST readily traps some radicals in the cell membrane. As previously reported, the terminal ring can scavenge radicals outside and inside of the cell membrane, and the double bond in the middle removes high-energy electrons [2, 9, 10]. The intracellular antioxidant activity of AST can be additionally achieved by the expression and regulation of oxidative stress response enzymes such as HO-1, an oxidative stress marker, and various antioxidant enzymes such as SOD2, catalase, and GPX1. These serial processes also activate the Nrf2/HO-1 antioxidant pathway by generating small amounts of ROS. Therefore, the unique features of the AST derive significantly high antioxidant activity by activating the antioxidant defense system of cells through upregulation of Nrf2 as well as direct radical scavenging [1]. Thus, the advantageous benefits of AST, which have an excellent effect on a variety of biological activities, can be utilized to prevent oxidative stress-related diseases, chronic inflammation, and treatment and prevention of skin diseases [11].

When human skin is continuously exposed to environmental factors such as UV radiation and ozone, the cellular level damages were naturally induced by the accumulation of excessive levels of ROS, and aging occurs [12]. ROS also plays a critical role in the normal wound healing response, and maintaining moderate levels is essential. Oxidative stress, such as the generation of ROS, is highly involved in various pathological conditions [13], such as wound healing, inflammation, and biocompatibility of transplantation materials [14]. ROS are naturally generated during normal metabolic processes, but when they are not properly regulated or accumulated excessively, various cellular damage occurs. When the body’s metabolic and antioxidant defense mechanisms are slowed down or problematic due to aging or pathological conditions, with a decrease in endogenous enzymes, more ROS is produced than can be eliminated by endogenous enzymes. Therefore, an increase in inflammatory cytokines by ROS can lead to chronic inflammation, which increases the risk of various diseases [15]. Therefore, it is important to balance ROS levels by appropriate regulation. In this respect, the ROS scavenging ability of AST can be one of the promising candidates as an excellent antioxidant application to regulate redox balance in wound healing [10]. Additionally, AST penetrates the skin, removes ROS on the skin surface, protects the dermal layer from ROS-related damage, and reduces inflammation induced by UV light and the like to help the skin regenerate. As for the notable capability, it reduces ROS at the cellular level through strong antioxidant action, and superficially minimizes the effects of ROS, as well as helping skin and tissue regeneration. The application of antioxidants can rapidly improve damage and wound healing caused by oxidative stress. Although AST is a powerful antioxidant, it is highly susceptible to heat, light, oxygen, and other environmental factors, which degrade easily during preparation and storage. It also has the disadvantage of low bioavailability in vivo applications due to lipophilicity [10]. For that reason, complexes such as encapsulation or used as a delivery system by increasing the bioavailability of AST are required, and it is necessary to improve bioavailability and delivery systems. Therefore, innovative AST-modification strategies will be needed to overcome these molecular activity limitations in the tissue engineering and wound therapeutic field.

Graphene is a two-dimensional (2D) carbon material that has been explored in biosensors, drug delivery systems, cell culture platforms, and electronic devices using its superior mechanical, electronic, and thermal properties [16–18]. An inherent 2D layer characteristic feature of graphene consisting of a unique layered carbon structure has shown exceptional physicochemical properties, and its excellent optical, electrical, and physicochemical properties have made it a promising tool in the biomedical field [19]. The arrangement of strong C–C bonds in the plane, aromatic structure, presence of free electrons, and surface reactive sites exhibited excellent biological properties [20]. Graphene-based materials may include radical adduct formation at the sp^2^ carbon site, which delocalized spin across the conjugated graphene backbone and leads to the destruction of radicals after the formation of a second adduct, which is achieved via electron transfer, hydrogen donation from functional groups, chelation of transition metal ions, and inhibition of radical generation [21]. Therefore, functionalized graphene-based materials can also eliminate ROS. Graphene oxide (GO) forms a dense structure with a layered sheet of carbon atoms and is composed of hydroxyl and epoxide functional groups on the surface and carboxyl groups on the edges [18]. GO is the oxidized form of graphene, produced by rigorous oxidation in an aqueous suspension. It also has colloidal stability with a negative surface charge. The basal surface of GO sheets is uncharged but contains hydroxyl and epoxide functional groups and is hydrophobic; therefore, stabilizing the hydrophobic molecule or creating an amphiphilic sheet-like molecule [20, 22]. Because the surface of GO contains functional groups such as epoxy and oxygen functional groups, such as hydroxyl, carboxyl, and carbonyl, it exhibits advantageous properties for tissue engineering and regeneration [23]. Due to its high biocompatibility, GO continues to be used to enhance cellular behavior for the development of numerous tissue engineering applications [24]. On this, bacterial infection and oxidative stress are critical issues in wound closure and healing processes because they delay wound healing and often lead to serious complications. Therefore, an appropriate dressing with intrinsic antibacterial activity as a barrier against external bacterial infection is critical in wound healing. GO and graphene-based nanocomposites can be one of the most promising materials as wound healing mediators because they improved antibacterial activity and biocompatibility with cells [25]. The presence of carboxyl (–COOH), carbonyl (–C = O), and hydroxyl (OH) groups on the edges of GO promotes interaction with biomolecules and induces the death of bacteria. Particularly, the edge of GO sheets can physically damage bacterial membrane, and inactivate bacteria because of leakage of the intracellular matrix [26]. RGD peptide is a major cell adhesion peptide as a bioactive ligand that improves cellular behavior by regulating cell adhesion, migration, proliferation, and differentiation [27]. Furthermore, it is known as a primary recognition motif of ECM protein containing three sequential amino acids, and the functionalization of the RGD peptide can improve cell adhesion and growth through surface-mediated interactions between cells and substrates [28]. As recently reported [29], RGD-functionalized GO nanosheets were found to be highly useful nanomaterials for biomarkers and a functional biomimetic sensor, in which the RGD peptide can be covalently bonded to the GO surface. Since the nanostructured surface enhances bioactivity through a large surface area, the morphological effect in the RGD-modified scaffolds significantly enhanced the cell adhesion of surrounding cells and tissues such as cell adhesion and proliferation [20].

Here, we report a facile route to produce a newly designed biomaterial consisting of GO nanocarrier and potent antioxidant of AST in a straightforward manner. Specifically, an ultrathin scaffold in a form of molecularly surface-grafted GO films was prepared to support an affinitive AST effect of removing active oxygen, in which GO sheets were combined together with exceptional chemical and physical properties as a multifunctional component. Firstly, we evaluated the cotreatment of GO-AST solution and demonstrated to determine the antioxidant effect with a radical scavenging activity because ROS is an important parametric indicator in the wound healing process and involved in the balance and regulation of the intercellular activities by generation and elimination. In addition, GO/AST film with RGD peptide was presented by a simple synthetic route, exhibiting excellent antibacterial effects by physicochemical damage. Along with this excellent antibacterial effect, the GO/RGD/AST film inhibited H_2_O_2_-induced intracellular ROS and suppressed the toxicity of LPS-induced cells by tightly binding to the biocompatible nanocarrier, thereby representing strong antioxidant and anti-inflammatory effects. Based on promising antioxidant and antibacterial reactions, we envision that the new concept of the GO nanocarrier-assisted bioabsorbable AST will be helpful in the treatment to prevent skin diseases or wound healing for the development of a new substance as a therapeutic strategy [30], ranging from drug delivery to tissue engineering [31].

## Materials and methods

### Surface modification of the glass substrate

To enhance the adhesion of individual GO sheets onto glass surface during flow-enabled self-assembly **(**FESA) process, the glass surface was sequentially modified using the hydroxylation process and the formation of a self-assembled 3-aminopropyltriethoxysilane (APTES) monolayer; the glass substrate was cut to a size of 1.4 × 1.4 cm^2^. In the first step, the glass substrate was cleaned and hydrophilized on its surface by immersing in piranha solution (H_2_SO_4_/H_2_O_2_, 3:1 v/v) for 1 h at room temperature. The piranha–treated glass substrate was then washed several times with deionized (DI) waters and dried using N_2_ gas. Then, the hydroxyl-terminated glass was immediately immersed into the prepared ATPES solution (Acetone/DI water, 5:1 v/v) for 2 h at room temperature, forming a self-assembled APTES monolayer with the amine-terminated group. The APTES-modified glass substrate was thoroughly rinsed with acetone to remove unbound silane residues and then dried with N_2_ gas.

### The fabrication of GO thin film via Flow-enabled self-assembly (FESA) process

GO was obtained via a modified Hummer method as reported previously [32]. The GO sheets were dispersed in DI water at a concentration of approximately 2 mg mL^−1^ to uniformly deposit on a glass substrate using the FESA process. The GO suspension (c = 2 mg mL^−1^) was then injected at the capacity of 20 μL between a confined geometry consisting of a stationary upper blade and a movable lower substrate fixed on a systemically controlled translational stage. Subsequently, GO thin film was uniformly formed onto the APTES-modified glass surface by continuously moving the lower substrate with a constant velocity of 5 mm s^−1^ and deposit number of 50 cycles.

### Multiple surface functionalization of GO thin film using RGD peptide and AST

The stacked GO sheets on the glass substrate were functionalized with two different biomolecules, such as RGD peptide and AST. RGD peptide was covalently bonded on GO thin film as previously reported for form RGD-GO complex [27]. First, 200 μL aqueous solution consisting of 260 μM of N-(3-dimethylaminopropyl)-N′-ethylcarbodiimide (EDC) and 520 μM of N-hydroxysuccinimide (NHS), were dropped onto the GO thin film for 1 h. Following that, the activated GO thin film was carefully rinsed with DI water. Then, 200 μL of RGD peptide solution (1 mg mL^−1^) was dropped onto GO thin film and incubated for 4 h. Meanwhile, AST-dimethyl sulfoxide (DMSO) solution was prepared at the concentration of 1 mg mL^−1^ and dropped onto RGD-functionalized GO thin film. After incubation for 4 h, unbounded AST molecules were rinsed using DMSO solution. To fabricate the ultrathin GO film-based wound dressing patch, 1 wt.% hyaluronic acid (HA) solution was spin-coated onto GO/RGD/AST film formed on a glass substrate at 1000 rpm for 20 s. Baking was performed on a hot plate at 100 °C for 15 min to remove the solvent (i.e., water) in HA film. After the HA film was completely dried, the GO/RGD/AST film was carefully peeled off with the HA carrier film from the glass substrate.

### Material characterization

Fourier-transform infrared spectroscopy (FT-IR) analyses were used to confirm the presence of diverse functional groups of materials and the structure of the molecule. FT-IR identified chemical bonds in a molecule by producing an infrared absorption spectrum; thus, FT-IR spectra were obtained through the absorption of electromagnetic waves in the infrared range, which is the specific energy. The FT-IR spectra of GO and AST were measured in the range of 4000–400 cm^−1^ using attenuated total reflectance (ATR), which is recorded using an FT-IR spectrophotometer (Spectrum GX, PerkinElmer Inc., Boston, M.A., U.S.A.). Raman spectra of GO, GO/RGD, GO/AST, and GO/RGD/AST films were separately characterized using a Raman spectrometer (wavelength = 532 nm, NS220, Nanoscope Systems, Daejeon, South Korea). The morphological structure of the GO, RGD peptide, and AST were characterized using transmission electron microscopy (TEM, TALOS F200X operated at 80 kV). GO film, GO/RGD film, GO/AST film, and GO/RGD/AST film were observed using scanning electron microscopy (SEM, Carl Zeiss AG-SUPRA 25 VP, 5–10 kV). Surface topography of GO film, GO/RGD film, GO/AST film, and GO/RGD/AST film was analyzed using atomic force microscopy (AFM, Park Systems, XE-100, South Korea) in non-contact mode. The tensile strength of the GO/RGD/AST-based wound dressing patch was evaluated by a texture analyzer (CT3, Brookfield Engineering, U.S.A.). The freestanding GO/RGD/AST/HA film was cut in size to 20 × 10 mm^2^. Next, the specimens were tightly clamped at intervals of 10 mm on both ends of the film. As pulling the upper clamp at the testing speed of 0.1 mm s^−1^, the maximum tensile strength and elongation of the AST-loaded GO/HA wound dressing were measured.

### Antioxidant assay

2,2′-azino-bis(3-ethylbenzothiazoline-6-sulfonic acid) (ABTS) and 2.2-diphenyl-1-picrylhydrazyl (DPPH) are relatively stable radicals and are commonly used to determine the antioxidant activity of natural substances [21, 33]. The ABTS radical was activated by mixing 7.4 mM ABTS and 2.6 mM potassium persulfate before reacting the mixture in the dark at room temperature for more than 18 h. 1 mL ABTS^+^ radical solution was added to GO, AST, and GO-AST solutions and reactive at room temperature for 30 min. Absorbance was determined at 734 nm using a UV–Vis spectrophotometer.

DPPH radical was prepared by dissolving in equal volumes of methanol and distilled water, at a concentration of 0.08 mg mL^−1^. 1 mL of the prepared DPPH radical solution was added to GO, AST, and GO-AST solutions. Then, absorbance at 540 nm was determined using a microplate reader (SpectraMax® 340, Molecular Device Co., Sunnyvale, CA). The scavenging ability of the radical was calculated using the following equation:

$$\mathrm{Scavenging}\;\mathrm{ability}\;(\%)=(\mathrm{control}-\mathrm{sample})/(\mathrm{control})\times100$$

### Antibacterial assay

In the experiment, antibacterial was used including *Escherichia coli* (*E. coli*, ATCC 8789) as a Gram-negative and rod-shaped bacterium, and *Staphylococcus aureus* (*S. aureus*, ATCC 27,217) as a Gram-positive and round-shaped bacterium. Bacteria were cultured in the Luria − Bertani (Difco LB, BD Biosciences) broth medium at a stable temperature of 37 °C for 24 h. The antibacterial activity of GO, GO/RGD, GO/AST, and GO/RGD/AST films were evaluated using the drop test method. The optical density at 600 nm represents the density of the bacteria [34]. The cultured bacterial (*E. coli* and *S. aureus*) were adjusted to be 1.0 optical density value at 600 nm, and 1 mL of the culture was centrifuged at 6000 rpm for 5 min to obtain bacterial pellets. Then, the pellets were resuspended in 1 mL phosphate buffered saline (PBS), 0.01 mL of bacterial cells dropped on film were exposed for 30 min at room temperature before their surface was washed with 5 mL PBS, then incubated at 37 °C for 24 h after spreading, and colonies were identified.

### Cell culture and cell viability

L-929 murine fibroblast cells were cultured in a DMEM medium containing 10% fetal bovine serum (FBS) and 1% penicillin–streptomycin at 37 °C with 5% CO_2_, the cells were passaged three times weekly. The cells were seeded on the GO/RGD/AST film and were incubated for 24, 48, and 72 h. The cell viability was measured using a reagent (WST-1) (Ez-Cytox; iTSBiO, Seoul, South Korea) to measure mitochondrial dehydrogenases in viable cells as a colorimetric assay for cell quantification. WST regents of 10% were added to each well, and absorbance was determined at 450 nm using an ELISA reader (SpectraMax® 340, Molecular Device Co., Sunnyvale, CA).

### Oxidative stress induction by H_2_O_2_ and intracellular ROS analysis in L-929 cells

Hydrogen peroxide (H_2_O_2_) was used to establish an intracellular oxidative stress model [35]. ROS were analyzed using a CM-H_2_DCFDA molecular probe (Invitrogen, C.A., U.S.A.), which was measured on the basis of fluorescence because of the conversion of fluorescent DCF in the presence of ROS as nonfluorescent H_2_DCFDA dye readily penetrated in the cells [36], which is a commonly used system for directly evaluating cellular redox states, and is a typical indicator of oxidative stress [37, 38]. The L-929 cells were seeded on the GO, GO/RGD, GO/AST, and GO/RGD/AST films. They were incubated for 24 h and treated with H_2_O_2_ (200 μM) for 2 h. Then, the cells were treated with DCFDA (5 μM), which is a commonly used ROS marker at 37 °C for 30 min in dark conditions. DCFDA will enter the cells if ROS is present and oxidized and cleaved with DCF to produce green fluorescence. We obtained fluorescence micrographs, and the degree of fluorescence was quantified using ImageJ software.

### Live and dead measurement of LPS-induced L-929 cells

The L-929 cells were seeded on the GO, GO/RGD, GO/AST, and GO/RGD/AST films with a density of 1 × 10^5^ cells mL^−1^. They were incubated for 24 h, and the medium was replaced with a fresh medium, and then stimulated using Lipopolysaccharide (LPS) treatment (1 μg mL^−1^) for 24 h. Cell viability and imaging were confirmed using live and dead cell assay (LIVE/DEAD™ Viability/Cytotoxicity Kit, Invitrogen™). First, remove the medium from the cells, then the dye mixture of the LIVE/DEAD assay kit (add 5 μL of calcein-AM and 20 μL of ethidium homodimer-1 to 10 mL PBS) was added and incubated for 30 min at room temperature in the dark. Then, the cells were visualized using a fluorescence microscope. Live cells appeared fluorescent green and dead cells fluorescent red. Fluorescence imaging was performed on a CELENA® S Digital Imaging System with EGFP (excitation 470 nm, emission 530 nm) and RFP filters (excitation 530 nm, emission 605 nm). The fluorescence images were analyzed and quantified using the ImageJ software.

### Statistical analysis

The collected data from independent experiments were quantified and analyzed for each variable. For each result, three or four independent experiments were performed, and the quantitative data are expressed as the mean ± standard deviation (SD). Statistical analysis was carried out using a one-way analysis of variance (ANOVA), which compares three or more levels within one factor, followed by a Bonferroni and Tukey test for multiple comparisons. A value of *p* < 0.05 was considered to be statistically significant.

## Results

### The preparation of ultrathin GO film-decorated with RGD/AST

**Fig. 1:**
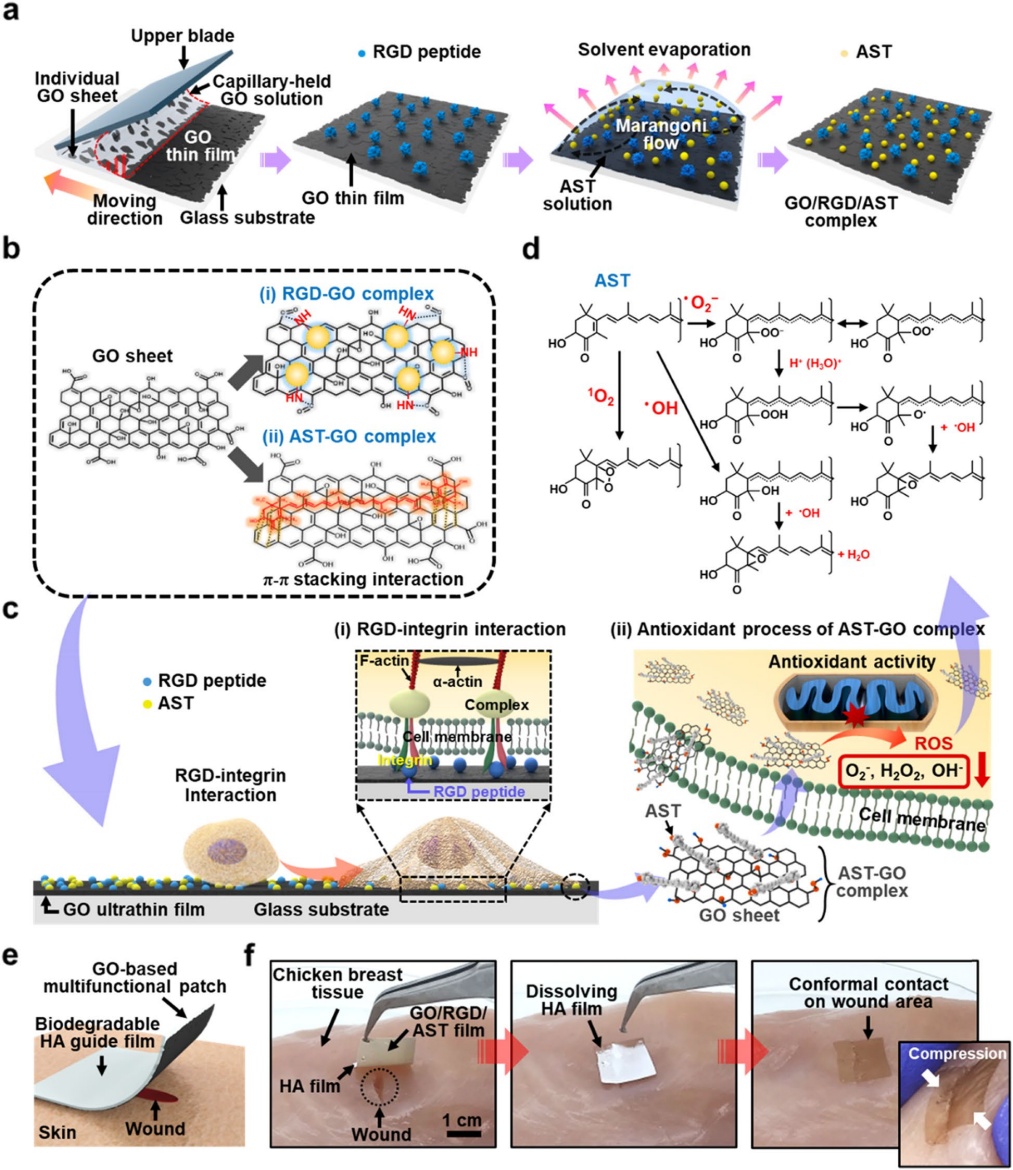
**a** The schematic illustration of the fabrication process on ultrathin GO film by flow-enabled self-assembly and consecutive RGD and AST deposition process. **b** Biomolecule-functionalized GO sheets using RGD (i) and AST (ii). **c** The conceptual drawing on the surface-mediated cell functions, enhanced by RGD-integrin interaction (i) and the antioxidant activity of the AST-GO complex (ii). **d** The possible antioxidant mechanism through the interaction of AST with ROS. **e** The conceptual image on GO-based patch-type wound dressing. **f** Transfer printing of the GO/RGD/AST patch directly onto the chicken breast; the inset image in the right panel shows a typical example of maintaining conformal contact of the ultrathin wound dressing under a harsh compression

Figure 1a illustrates the sequential process to engineer the GO-based bioabsorbable multifunctional ultrathin film (i.e., GO/RGD/AST film) for enhanced cell adhesion and antioxidant activity. As a form of 2D nanosheets, GO was selected as a nanocarrier because it enables multiple interactions with diverse proteins by containing abundant oxygen functional groups, such as hydroxyl, epoxy, and carboxyl groups. In our experimental scheme, the following subsequent steps were used to control evaporative self-assembly to design multifunctional biointerface: i) uniform deposition of individual GO nanosheets dispersed in colloidal solution using flow-enabled deposition process in confined geometry and ii) chemical modification with biomolecules (i.e., RGD and AST) on the ultrathin GO film through the careful control of droplet evaporation. First, the flow-enabled self-assembly (FESA) was performed on a substrate by trapping a drop of GO solution between the upper blade at an angle of 30-degree and the lower flat substrate (i.e., glass); this method is an extremely simple and effective strategy to uniformly deposit the GO nanosheets, as previously reported [32]. Before proceeding with the FESA process, the glass substrate surface was hydroxylated by piranha solution treatment and subsequently introduced APTES monolayer with NH_2_ terminal group to improve the adhesion of the deposited GO nanosheets with the substrate. Next, a sessile drop of the GO solution (c = 2 mg mL^−1^) was injected in the gap between the upper blade and the glass substrate (volume = 15 µL), where a meniscus of GO solution was firmly fixed and trapped by capillary force. Then, the lower substrate, placed on a motorized translational stage, was repetitively traveled to drag the meniscus at the velocity of 5 mm s^−1^ with 50 cycles; in this geometry, the upper blade was held stationary state. Consequently, the self-organization of the GO nanosheets was crafted by spontaneous solvent evaporation as the meniscus repetitively moved in a programmable manner. As a result, an ultrathin GO film on a glass substrate was uniformly formed by the stacking of the GO nanosheets on a substrate.

As a next step, the RGD peptide was further incorporated into the surface of the prepared planar GO film by the controlled evaporation of the solution droplet (middle panels in Fig. 1a). Since the outward radial capillary flow was driven toward the edge of the contact line, the highest evaporation rate was observed at the droplet edge as the solvent gradually evaporated [39]. However, Marangoni flow spontaneously is involved in the surface tension-driven gradient recirculation, surrounding the droplet surface. In this way, the RGD peptide with terminal amino functional groups was firmly immobilized onto the GO surface via a condensation reaction with carboxyl activation using NHS/EDC complex reagents as presented in Fig. 1b (i) [40]. Similarly, AST molecule was also applied on the GO/RGD film using a solution droplet deposition. AST is a π-conjugated polyene biomolecule that consists of a skeletal structure, such as a polyene chain and two terminal rings bearing two hydroxy substituents. From a structural point of view, the multi-stacked GO film can be used as an underlying support surface for anchoring AST molecules due to the π–π stacking interactions with GO (i.e., hexagonal carbon networks) and the aromatic ring in AST. At the same time, a strong hydrogen bond can be involved between hydroxyl (–OH) groups on AST and the –OH/–COOH groups on the GO surface, respectively. In Fig. 1b (ii), a conjugated complexation of AST-GO through π–π interaction and hydrogen bonding is schematically presented.

The main conceptual approach was derived from the RGD-integrin interaction and the antioxidant activity of the AST-GO complex; the enhanced surface-mediated cell functions are illustrated in Fig. 1c. As an integrin-binding motif, the RGD peptide was advantageously adopted to enhance the binding affinities of the cells on the ultrathin GO film as illustrated in Fig. 1c (i). Also, as schematically illustrated in Fig. 1c (ii), the AST-GO complex may positively affect cells by sustainably supplying antioxidant equipped in nanocarrier (i.e., GO), suppressing the production of oxidative stress. Since the chemical structure in AST contains conjugated double bonds in the polyene chain and polar hydroxyl (–OH) and keto group (–C = O) at both ends of the polyene chain [41], the unique GO-AST efficacy was studied in controlling ROS reaction associated with scavenging hydroxyl radicals and superoxide, inhibiting multiple oxidative chain reactions, as summarized in Figs. 1d and S1 [42]. Moreover, a strong antioxidant effect can also be exerted on conjugated double bonds that donate electrons by reacting with free radicals [43]. Thus, we believe that a planar configuration thin film of the GO/RGD/AST promises to achieve higher levels of antioxidant efficacy and ensured cell functions for stable chemical antioxidant transport across physical barriers.

As a conceptual example in our approach, GO/RGD/AST-based wound dressing can be suggested as illustrated in Fig. 1e, which exhibits an idea of patch type application. The ultrathin GO film decorated with RGD and AST molecules can be transferred to the biocompatible polymeric carrier film and used in the skin wound healing processes. In Fig. 1f, the sequential digital images display an applied GO/RGD/AST-based wound dressing on the chicken breast surface with a support layer of HA film; the details of the transfer printing process for wound dressing preparation are described in Fig. S2. In this scheme, the direct contact of GO/RGD/AST-based wound dressing patch to a wound site in conformal configuration, and then the carrier HA film was gradually gelated and dissolved by water. The supportive HA is also advantageous with excellent moisture retention in this application because the biodegradable HA is a natural polysaccharide generated during the proliferation of fibroblasts at the wound repair stage. As seen in the right panel in Fig. 1f, the ultrathin nature of the GO film and tightly bonded film structure ensures stable conformal contact onto the wound site under harsh mechanical constraints such as compression and stretching without physical degradation of the wound dressing film. As clear guidance in this experimental approach, the maximum tensile strength and elongation of the AST-loaded GO/HA-based wound dressing patch (*t* = 67.5 ± 8.5 µm) were measured to be ~ 21.3 kPa and ~ 7.1%, respectively, as presented in Fig. S3. In general, the mechanical properties of wound dressing patch can affect the growing fibroblasts, involved in the wound healing process, and it has been known that the matrix stiffness roughly from ~ 1.3 to ~ 23 kPa enhances the formation of stress fibers with an increased proliferation of fibroblasts [44]. Therefore, within our experimental condition, it was confirmed that the mechanical strength of AST-loaded GO/HA-based wound dressing can be worth being used in promoting wound healing and supporting tissue repair. With a limited condition for in vivo experiments, an animal test for wound healing could not be performed at this stage. However, in the following section, we carefully evaluated a set of properties under in vitro conditions for the antioxidant and antibacterial effect of GO/RGD/AST films on wound healing materials.

### Characterization of GO/RGD/AST film

**Fig. 2:**
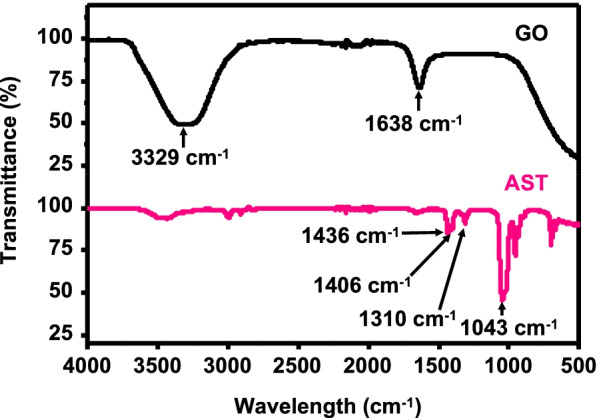
FT-IR spectra of GO and AST. The peaks for GO (3329 and 1638 cm^−1^) and AST (1436, 1406, 1310, and 1043 cm^−1^) recorded using an FT-IR spectrophotometer and the ATR method in the range of 4000–400 cm^−1^

To confirm the presence of diverse functional groups of GO and AST, FT-IR analyses were used as shown in Fig. 2. Various surface functional groups appeared from GO, such as carboxyl, hydroxyl, and epoxy groups [45]. FT-IR spectrum of GO reveals the characteristic bands of C = C stretching and the N–H bending around at 1638 cm^−1^, representing a carbon–carbon double bond, and main peaks were observed around 3329 cm^−1^ because of O–H and N–H stretching. The FT-IR spectrum of AST displays strong absorption bands at 1043 cm^−1^ due to C–O and C–N stretching. Other peaks were observed around 1436, 1406 cm^−1^ (C–C stretching), and 1310 cm^−1^ (C–O and C–N stretching).

**Fig. 3:**
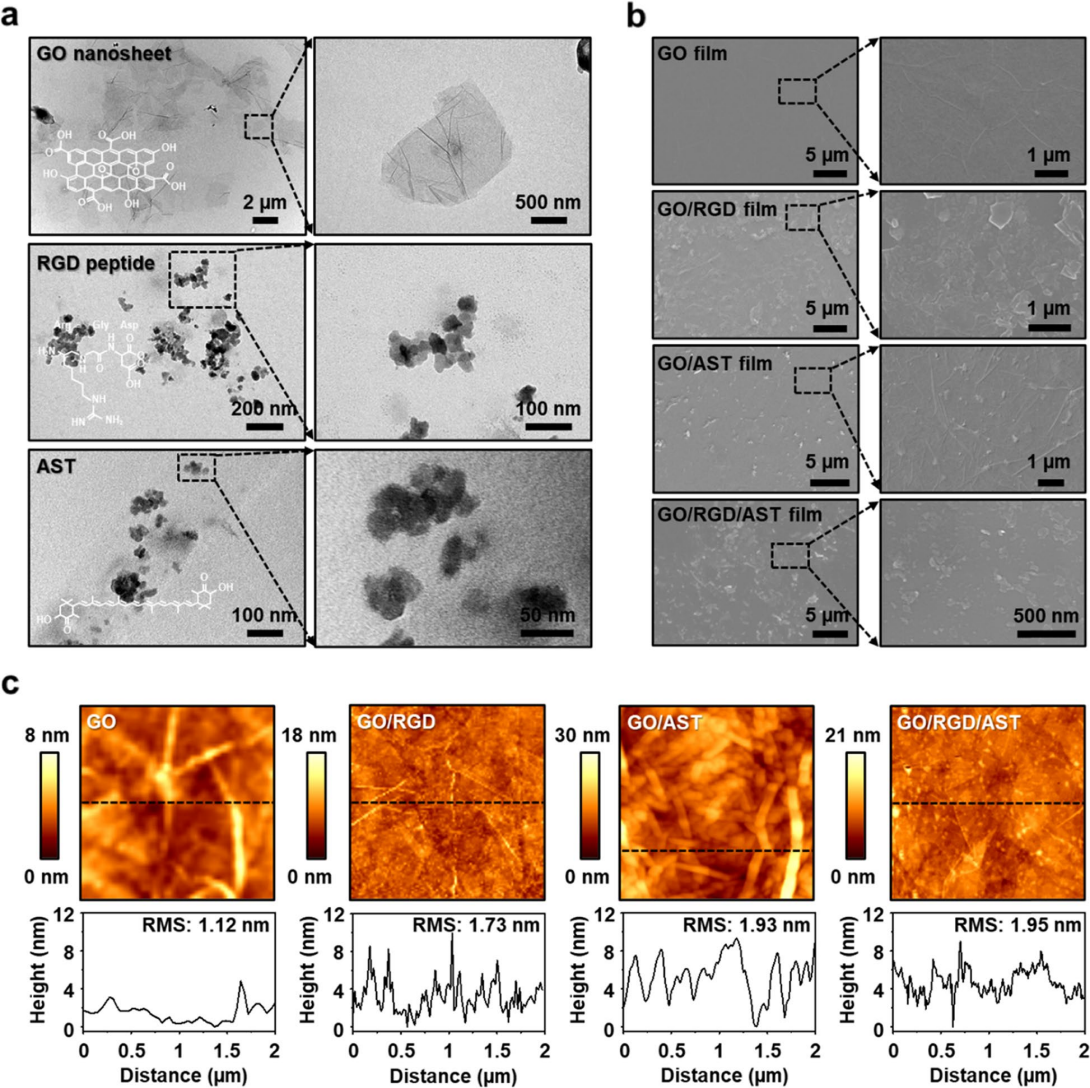
**a** TEM image of GO nanosheets, RGD peptide, and AST. **b** Highly magnified SEM image representing morphological features of GO, GO/RGD, GO/AST, and GO/RGD/AST films. **c** Topographical AFM image of GO, GO/RGD, GO/AST, and GO/RGD/AST films

The characteristic morphological features on the surface of nanocarrier-enabled GO in an ultrathin film form were analyzed by TEM, SEM, and AFM. As shown in Fig. 3a, each colloidal sample was characterized by TEM measurement. It was confirmed that the GO nanosheet was highly soluble in water and alcohol because it was present in the form of bonds with hydroxyl groups (–OH) and epoxy groups (–O–) on the surface and carboxyl groups (–COOH) on the edges. The RGD peptide particles prepared for this experiment were distributed in uniformly aggregated forms. The AST dispersed in solution was observed in a spherical shape and slightly aggregated, similar to the RGD case. Each molecule immobilized on GO nanosheets was carefully measured to ensure complexation before being used in the deposition process (Fig. S4). The relatively uniform size distribution was in the range of ~ 15–25 nm for the RGD peptide. Interestingly, however, the AST particles loaded on the GO nanosheets were found to be in a wide range of ~ 30–50 nm, similar to that observed in colloidal solution. In the following, we used SEM to measure the surface in a sequentially performed FESA and droplet evaporation method (Fig. 3b). Some nanoscale wrinkles were observed as a stacking layered structure of GO film. When the ATS and RGD were deposited separately on the GO thin film on a glass substrate, the SEM images indicated similar but slightly different surface morphologies; the ATS coated samples displayed a more uniform surface that the RGD case, which might be attributed to the compatibility of the chemical structure between GO and AST. In contrast, the measured SEM image from the GO/RGD/AST film confirmed relatively a more flattened surface, compared to the GO/RGD case where the particles are aggregated on the stacked GO surface. Instead, the GO/RGD/AST film was crafted with high uniformity, similar to the GO/AST films. The additional AFM measurements provided more precise information on the surface topographies, as presented in Fig. 3c. The AFM images and corresponding root mean square (RMS) surface roughness indicated good agreement with the measured SEM images. In the case of the ultrathin GO film, the surface roughness was found to be ~ 1.12 nm with highly uniform features with nanoscale wrinkles. Thus, in the cases of the GO/RGD and GO/AST, the higher levels of the RMS numbers were measured to be ~ 1.73 and ~ 1.93 nm, respectively. However, the GO/RGD/AST films were valued at ~ 1.95 nm in a similar range to the GO/AST case. An additional analysis was performed to qualify the complexation of the biomolecules (i.e., RGD and AST) on the ultrathin GO film, the prepared samples were sequentially investigated by Raman spectroscopy, as presented in Fig. S6. As well-known, the typical Raman spectrum of GO film shows two characteristic peaks at 1346 cm^−1^ (D bands) and 1598 cm^−1^ (G bands), corresponding to sp^3^ structural defects in the GO basal planes and the vibration from the sp^2^ carbon lattice, respectively [46]. Compared to the original GO film, strong peaks at 1008 cm^−1^ (C–H bending), 1157 cm^−1^ (C–H stretching), and 1519 cm^−1^ (C = C stretching) appeared from the GO/AST films, in which the major main peaks are in agreement with the previous report [47]. In the case of the GO/RGD film, several characteristic peaks were featured due to the vibrations of amide bonds of the polypeptide structures at 1480 cm^−1^ and the presence of the aromatic amino acid at 1280 cm^−1^ and 1675 cm^−1^ [48]. The final measurement of the Raman spectra for GO/RGD/AST film confirmed a successful surface-enabled complexation to prepare the following series of experiments.

### Antioxidant activities of GO and AST with the acellular model

**Fig. 4:**
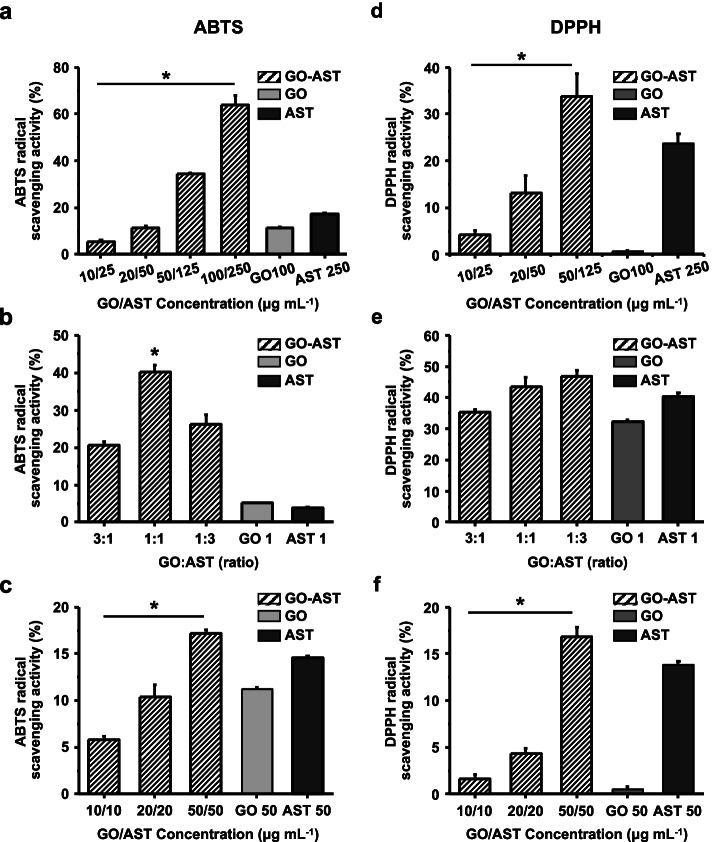
Evaluation of antioxidant activity against ABTS and DPPH radical species, compared to the untreated control groups. **a** ABTS radical scavenging activity for each concentration of GO, AST, and GO-AST solution (GO; 10, 20, 50, and 100 µg mL^−1^ and AST; 25, 50, 125, and 250 µg mL^−1^). **b** ABTS radical scavenging activity according to the ratio of GO and AST. **c** Concentration-dependent antioxidant activity at the same ratio (GO and AST; 10, 20, and 50 µg mL^−1^). **d** DPPH radical scavenging activity for each concentration of GO, AST, and GO-AST solution (GO; 10, 20, and 50 µg mL^−1^ and AST; 25, 50, and 125 µg mL^−1^). **e** DPPH radical scavenging activity according to the ratio of GO and AST. **f** Concentration-dependent antioxidant activity at the same ratio. GO and AST; 10, 20, and 50 µg mL.^−1^ (*: *p* < 0.05)

Antioxidant activity can be evaluated by radical scavenging assays, according to the reactivity of free radicals and antioxidants, ABTS and DPPH assays are frequently used for these experiments [35]. ABTS^+^ radicals are dark blue–green, they react with strong antioxidants, their color fades to near transparent. After adding GO and AST by each concentration to ABTS radical solution reacting at room temperature for 30 min, absorbance was measured at 734 nm to compare with the control group without sample addition. The scavenging activity of ABTS radicals of GO (10, 20, 50, and 100 µg mL^−1^) and AST (25, 50, 125, and 250 µg mL^−1^) combination treatment increased in a concentration-dependent fashion, as presented in Fig. 4a. Especially, the apparent effect was increased in the combined treatment than the independent treatment cases when measured in the same concentration range. Among combined treatment GO and AST in a ratio of 3:1, 1:1, and 1:3, the highest ABTS scavenging activity was found at a ratio of 1:1 case (Fig. 4b). As a result of measuring each concentration level for 10, 20, and 50 µg mL^−1^ at a 1:1 ratio of GO and AST (Fig. 4c), it showed a concentration-dependent increase similar to the above result; combination treatment was more effective than a single treatment. These results indicate that the combined effect on ABTS radical scavenging activity was increased when GO and AST was treated together.

DPPH assay is a chemical method for determining the antioxidant capacity of natural compounds based on a decrease in absorbance during free radical scavenging reactions [49]. GO and AST was added to DPPH radical solution, reacted at room temperature, then the absorbance at 540 nm was measured. The measured values were derived from DPPH scavenging activity compared with the control group at which the sample was not added. When GO (10, 20, and 50 µg mL^−1^) and AST (25, 50, and 125 µg mL^−1^) were treated together, the levels of the DPPH radical scavenging activity were increased in a concentration-dependent trend. As shown in Fig. 4d, the effect was higher in the case of combined treatment rather than the single treatment. Consequently, the DPPH scavenging activity was confirmed by the GO and AST in the ratio of 3:1, 1:1, and 1:3. However, unlike the ABTS results, the highest DPPH scavenging activity was observed in the 1:3 ratio of GO and AST (Fig. 4e), suggesting that the ability of AST has a greater effect on the DPPH radical scavenging activity than GO. In addition, as a result of confirming the DPPH radical scavenging activity by treating with different concentrations at the same ratio, the activity of AST was higher than the GO treatment alone (Fig. 4f). Thus, AST plays an important role in the combinatorial state, notably, the evaluations of the ABTS and DPPH radical scavenging activity revealed the dual treatment of GO and AST markedly elevated the positive responses, compared to the respective single treatment tests.

### Antibacterial activities of GO/RGD/AST film

**Fig. 5:**
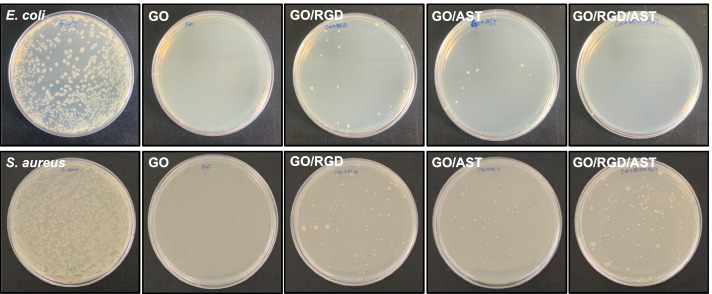
Digital images of agar plate colonies for each sample. Antibacterial effect against *E. coli* and *S. aureus* tested with GO, GO/RGD, GO/AST, and GO/RGD/AST films

Our proposed GO/RGD/AST film (i.e., film-type AST-decorated GO-based nanocarrier) may effectively prevent bacterial invasion and reduce the risk of infection as a physical barrier. In our experiment, the antibacterial effect was evaluated by the presence or absence of colony formation using *E. coli* and *S. aureus*, which are the most typical bacterium of Gram-positive and negative. As shown in Fig. 5, the colony formations were significantly reduced in GO, GO/RGD, GO/AST, and GO/RGD/AST films, compared to the control groups for *E. coli* and *S. aureus* alone. Notably, the slightly elevated sharp edges of the GO film can physically damage to form pores by the destruction of protein bonds on the bacterial membrane, which causes cytoplasmic leakage and destroys the membrane; the apparent colony formation (i.e., bacterial death) for inhibiting *E. coli* and *S. aureus* growth suggests a high antibacterial effect of GO/RGD/AST film.

### Inhibition of H_2_O_2_-induced ROS in L-929 cells cultured on GO/RGD/AST Film

**Fig. 6:**
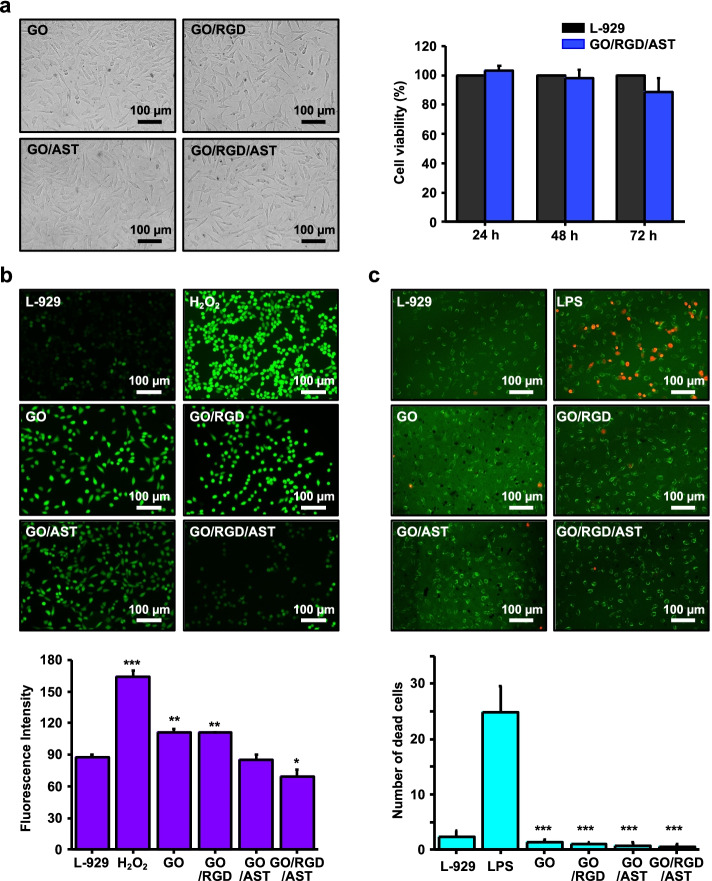
**a** Cell viability on GO/RGD/AST film according to each concentration as evaluated for 24, 48, and 72 h using WST-1 assay. **b** The inhibitory effect of ROS production on H_2_O_2_-induced L-929 cells in the GO, GO/RGD, GO/AST, and GO/RGD/AST films as analyzed using DCFDA fluorescence assay. **c** Live and dead cell assay on LPS-induced L-929 cells in the GO, GO/RGD, GO/AST, and GO/RGD/AST films (*: *p* < 0.05, **: < 0.01, ***: < 0.001)

For the evaluation of biocompatibility in vitro, L-929 fibroblast cells were seeded and cultured on the GO/RGD/AST films to determine cell viability. The cell suspension was applied to the prepared films by dropping, and WST-1 regent was added in an amount of 10% of the medium after incubating for 24, 48, and 72 h, followed by reaction at 37 °C for 2 h. Then, the cell adhesion and proliferation were confirmed using an optical microscope, and absorbance was measured at 450 nm. As presented in Fig. 6a, relatively good adhesion and growth were observed when the viability was well maintained up to 72 h, demonstrating that the GO/RGD/AST film is a sufficient biocompatible cell substrate. H_2_O_2_ is a redox signaling molecule that normally forms hydroxyl radicals and reacts within cells to produce various radicals, such as alkyl radicals. Short-term exposure to H_2_O_2_ can easily penetrate cells and generate endogenous ROS, inducing oxidative damage to cells [35]. A method to trigger intracellular oxidative stress by H_2_O_2_ is commonly used to evaluate the antioxidant activity of natural substances and is useful for observing the regulation of antioxidant molecules [49]. Thus, the L-929 cells suspension was seeded on the GO, GO/RGD, GO/AST, and GO/RGD/AST films, cultured for 24 h; ROS was induced by treating with H_2_O_2_ (0.2 mM) for 1 h. Subsequently, it was confirmed whether ROS was generated and inhibited through DCFDA staining for intracellular ROS marker. DCFDA reacts with intracellular ROS by cell-permeable diffusion and internalization and is converted to DCF with high green fluorescence [15, 35]. Under the blocked light condition at 37 °C for 30 min after the reaction with CM-H_2_DCFDA (DCFDA, 5 µM), DCFDA is oxidized with ROS in cells and converted into DCF, brighter fluorescence. As presented in Fig. 6b, the ROS level was obviously decreased in the cells cultured on GO/RGD/AST film, representing the lowest values (L-929; 87.88 ± 2.07, H_2_O_2_; 163.69 ± 6.2, GO; 111.27 ± 3.47, GO/RGD; 110.80 ± 0.48, GO/AST; 85.49 ± 4.45, GO/RGD/AST; 69.30 ± 6.46); ROS level was highest in the H_2_O_2_ treated group. Interestingly, when cultured on the GO/RGD/AST film, the measured cell area decreased right after H_2_O_2_ treatment but increased again (Fig. S6). The cells on GO/RGD/AST film appeared specifically reduced ROS level by ~ 42%, compared with the cells H_2_O_2_-treated alone, demonstrating a strong antioxidant effect at the cellular level by inhibiting intracellular ROS generation.

### GO/RGD/AST film reversed the LPS-induced cytotoxicity in L-929 cells

When oxidative stress is induced in the overproduction of ROS, the inflammatory response is prolonged. On this, ROS-induced inflammatory factors are deeply involved in LPS-induced cell death and damage, causing chronic inflammation [50, 51]. Therefore, it is necessary to proceed with the wound healing process through the inhibition and regulation of ROS and inflammatory factors. LPS is a component of Gram-negative bacterial cell walls and produces mediators of inflammation, of which nitric oxide (NO) is the most important mediator in inducing inflammation. Excessive generation of NO causes inflammatory diseases because it forms free radicals such as peroxide. Various inflammatory diseases and their complications are characterized by oxidative stress and inflammation. Furthermore, long-term progressed or uncontrolled abnormal inflammatory processes cause tissue damage and are responsible for numerous inflammatory chronic diseases. Therefore, the viability of cells in the induced inflammatory response through LPS was evaluated to confirm the cell protection role of GO/RGD/AST film in skin wound healing processes. As presented in Fig. 6c, GO/RGD/AST film decreased dead cells and reversed cell viability in LPS-induced L-929 cells. The results indicate that the inflammatory factors were reduced in the cells, which implied the application of GO/RGD/AST film could prevent the delay of the wound healing process.

## Discussion

Our main scheme of this study aimed to observe functionalized surface-mediated antioxidant and anti-inflammatory effects of AST-loaded ultrathin GO films. In cell responses with the demanding factors, the AST/RGD/GO film enhanced the adhesion and viability of the specific cell type that may be highly beneficial in wound healing or tissue regeneration. At the initial stage of the experiments, we hypothesized that the GO-based cell substrate combined together with RGD peptide and AST would provide highly beneficial cellular environments for modulating cell functions, including cell adhesion, migration, viability, toxicity, and apoptosis. In addition, we expected that uniformly assembled arrays of the GO film functionalized with RGD peptides would synergistically promote cell adhesion with the topographical features of the nanoscale GO film surface. As a set of the results, the internal flow fields and the interaction between suspended RGD peptide and the exposed GO surface played a critical role in the surface-mediated molecular assembly process. Thus, it was demonstrated that the mixed hydrodynamic flow of the RGD solution droplets leads to uniform assembly of the RGD molecules on the exposed GO surface in the convective evaporation of the solvent (i.e., GO/RGD film). Because the critical factor for surface-recognizing receptors on living cells is integrins, the provided key functions in the pericellular microenvironment (i.e., ECM) could mediate the cell adhesion, migration, and cell–cell interaction through bidirectional signaling pathways [52]. When the cells are close enough to contact the RGD ligands anchored on the planar GO surface, the integrin heterodimers are readily activated by undergoing conformational changes to promote cell affinity and allow interaction with proteins and signaling molecules on the cytoplasmic domain. After the activation of the integrin, the RGD ligand-integrin complexes can be formed at discrete locations on the cellular membrane. Subsequently, actin-based microfilaments bind to cytoplasmic proteins and organize actin filaments growing into bundles, maximizing interactions with cells. Indeed, the stable RGD ligand-integrin complex gradually matures into focal adhesions [53]. In another aspect from a scheme of cell metabolism, multiple oxidative chain reactions in mitochondria perform a pivotal role in generating adenosine triphosphate (ATP) through oxidative phosphorylation, producing large amounts of ROS (i.e., superoxide (O_2_^•−^), hydrogen peroxide (H_2_O_2_), and hydroxyl radical (OH^•^)). Because oxidative stress is progressively generated by an imbalance between ROS production and the operation of the antioxidant defense system, the ROS must be neutralized to maintain proper mitochondrial function (Fig. S7). Thus, oxidative damage in cell or tissue levels occurs by triggering the defect of mitochondrial DNA and cellular senescence when these antioxidant defense systems are not sufficient to control the generation of free radicals [54]. As noted, the AST acts as a powerful antioxidant agent to aid in neutralizing ROS generated in the cells, so thus the combinatorial physicochemical effects of GO with AST may reduce the oxidative stress accumulation in mitochondria and the dysfunction of mitochondrial metabolism.

On the preparation of the AST/RGD/GO film, the series of the results of surface analysis confirmed that the chemical interactions between RGD peptide and AST on the planar surface of GO film were also firmly established in our successive deposition system by the well-controlled binding affinity and comparable physical dimensional parameters (Fig. 3 and Fig. S5). After the deposition of RGD peptide and AST, the prepared GO surface adsorbed molecules by a surface-mediated chemo-physical deposition process. The ultrathin GO film produced from the FESA process appeared uniform surface morphology in a large area with a help of the programmable motorized stage and a tight chemical bonding of the GO nanosheets with the self-assembled monolayer layer on the glass substrate.

When the skin is damaged, oxidative stress is induced by the production and presence of free radicals at the wound sites. That is, normal antioxidant systems that improve wound healing by protecting skin and tissues from oxidative reactions have important implications in the field of tissue engineering. As previously reported [21], GO exhibited significantly high antioxidant activity in the form of scavenging hydroxyl and superoxide radicals, which might be mainly attributed to the scavenging activity of the pristine sp^2^ carbon domain of the basal surface, and this antioxidant activity can protect intracellular components from oxidation. Besides, AST provides powerful inhibitory and protective functions against free radicals and oxidation. As well known, the strong antioxidant activities of AST are originated from the hydroxyl and keto groups at the molecular terminals. The polyene chain of AST is capable of a strong antioxidant effect with a unique chemical structure that traps radicals [55], in which the long-conjugated double-bonded polyene possesses ROS removal capability by regulating the redox balance due to its inherent lipophilic and hydrophilic properties. Moreover, it exhibits higher antioxidant activity than other carotenoids such as β-carotene and lutein because of the presence of two oxygen groups, including hydroxyl (OH) and carbonyl (C = O) in each of the rings [56]. Consequently, as can be seen from the ABTS and DPPH assay of GO and AST, the synergistic combination of the AST and GO may provide unprecedented scenarios as new antioxidant material to those working in this nanobiotechnology field, thereby these materials can be considered as a strong candidate for an antioxidant platform for the treatment of various oxidative stress-mediated diseases.

The antibacterial effect of GO was recently explored on the basis of various mechanisms, interfacing with the bacterial membrane, due to physical damage, surface roughness, electrical potential, and electron transfer on the surface, which in turn induces pore formation as a major factor influencing the antimicrobial properties [57]. In particular, when exposed to sharp edges, pores are formed along with protein binding disruption, which ultimately leads to death by physical damage and destruction of the bacterial membrane. Furthermore, the electrical potential of the surface causes bacterial damage by electron transfer, and the degree of surface roughness directly interferes with the adhesion of bacteria to affect the antibacterial properties, so thus proper wettability has a significant antibacterial effect [58, 59]. Our results suggest the antibacterial activity of GO itself via physical damage because of the exposure of the basal surface and sharp edges on the ultrathin GO film. Moreover, the electrostatic repulsive force caused by the subtle charge differences between the bacterial membrane and GO/RGD/AST film surface might interfere with the adsorption of the bacteria. By this, the bacteria cannot maintain constant surface potential levels, which generally causes damage to the bacterial membrane [58]. The combinatorial effect of GO and AST could affect the surrounding bacteria and cause cell membrane damage, but their antibacterial efficacy subtly depends on the species of the microorganism and their relative cell size. Based on our experimental results, we confirmed that the AST-loaded GO films induced more cell membrane damage to Gram-negative *E. coli* than to Gram-positive *S. aureus* due to their different cell volumes and shapes. By the fact that the most Gram-positive bacteria are generally better able to protect cells than Gram-negative bacteria due to their thicker cell walls, we found that the colony formation and antibacterial effect were slightly different. Moreover, the rod-shaped *E. coli* with a larger cell volume was more easily attacked in the microenvironment with a larger contact surface area, compared with the round-shaped *S. aureus*, as previously reported [60–62]. As discovered recently [63], although AST itself derived a significant reduction of both bacterial growth and biofilm formation, the synergistic antibacterial effects of GO/RGD/AST film evidenced the active interactions with AST on GO film surface can efficiently penetrate the attached bacterial membrane. Therefore, we envision that the AST functionalization of the ultrathin GO film surface can be used for wound healing with antibacterial ability.

Oxidative stress in cells is caused by endogenous and exogenous factors with excessive production of ROS, which directly or indirectly affects the wound healing process. This consists of inflammatory, proliferative, and remodeling stages, and factors such as oxidative damage and bacterial infection have a harmful effect on the process. Chronic wounds are caused by the degradation of ECM proteins, functional weakening of dermal fibroblasts, and induction of abnormal inflammation. Thus, intracellular oxidative stress reduction is critical to maintaining fibroblast function, and the promotion of fibroblast migration/proliferation is the main feature for improving wound healing processes [49, 64]. The antioxidant defense system is greatly critical in regulating oxidative stress to maintain a balance between ROS generation and elimination, alterations, and damage to this can lead to an imbalance in ROS. Through this imbalance, biomolecules, such as DNA and proteins are oxidatively damaged, resulting in aging and various diseases. For this reason, antioxidant research on the carotenoids such as β-carotene, lutein, and AST has been extensively studied [35]. When oxidative stress is induced by continuous ROS generation or increased at high concentration, the main cause of wounds (e.g., NF-kB pathway) is initiated and leads to chronic inflammation. The surface-mediated interaction of L-929 cells on the GO/RGD/AST film could effectively inhibit intracellular ROS overproduction for redox balance regulation through the beneficial effects of AST and GO in the nanostructured film, ultimately restoring cell health. In this experiment, GO/RGD/AST film promoted the proliferation of L-929 cells and was helpful for scavenging intracellular ROS (Fig. 6). In other words, our developed GO-based ultrathin film can be viable a mediator for cell protection to maintain functions by regulating the secretion of proinflammatory cytokines and increasing resistance to oxidative stress. Therefore, although it can be limitedly used in treating diseases such as chronic wounds, neurodegenerative and cardiovascular diseases that are mediated by oxidative stress, its high biocompatibility and accessibility suggest promising materials system for wound dressing. ROS induces the production of inflammatory cytokines and is directly involved in immune responses, contributing to many inflammatory diseases [65]. The wound healing process generally includes an inflammatory process, but excessive inflammation can lead to chronic wounds. Thus, the wound healing period could be delayed or impaired because of abnormal inflammatory and immune responses, bacterial infection, and excessive ROS production. Generally, ROS levels at wound sites increases and then gradually decrease. Low concentrations of ROS are a pivotal role in normal wound healing response and regulation, and moderate ROS is closely related to the wound healing phase. However, ROS accumulation and oxidative stress by sustained ROS generation impairs the functions of dermal fibroblasts and keratinocytes, resulting in the modification and degradation of ECM proteins, which cause chronic inflammation and delay wound healing [50, 66]. Granulation tissue is a flexible, granular, bright red tissue observed during wound healing, and its basic component is fibroblasts, wound healing and recovery go through the process of granulation tissue formation and re-epithelialization [67]. Here, the proliferation and migration of keratinocytes and fibroblasts are the keys to the remodeling process, and providing microenvironments to prevent apoptosis is an effective method for wound healing and repair [59, 63–66]. Conclusively, our simple approach using AST surface-modified ultrathin GO film has a great potential to assist in wound healing by participating in a series of processes, such as cell proliferation, inflammation, angiogenesis, and remodeling.

A drug delivery system is a technology that efficiently supplies pharmacologically active substances and drugs to cells, tissues, or organs to minimize side effects while maximizing therapeutic efficacy [68]. In addition, newly developed biocompatible nanomaterials are one of the promising candidates as scaffolds for tissue engineering that regenerate damaged tissues and improve cell adhesion and growth [69]. In this context, because maintaining the redox equilibrium is an essential step in promoting tissue regeneration [70], a combination of GO/RGD/AST film can be a value-added strategy for the development of tissue-engineered scaffolds capable of antioxidant activity and surface-mediated drug delivery for wound healing. Within the constrained surface condition (i.e., GO/RGD/AST film), the release rate of the GO binding AST is presumably slower than that of direct treatment on the cells in the in vitro experiment. Similarly, as explored by Łupina et al. [71], the release of AST from the biopolymer carrier, such as cellulose/gelatin and octenyl succinic anhydride starch/gelatin films, appeared ~ 35–63% depending on the AST concentration; the increasing release level of AST also resulted in a gradual increase in the ABTS^+^ scavenging capacity and offered higher antioxidant activity. Although it is difficult to calculate the exact value on the release rate of AST in the GO-AST state, based on the correlation between the cumulative AST release and the antioxidant activity, the ABTS^+^ scavenging capacity from our experimental results inferred that the release rate of AST could be roughly estimated at over 40%. Because it is important to maintain and regulate optimal ROS levels, the GO/RGD/AST film can be a promising candidate for the wound dressing material primarily due to its antioxidant, anti-inflammatory, and antibacterial properties, as presented earlier. The multifunctional benefits of this planar film material also are expected to contribute to the maintenance of redox balance and to provide a positive cell-responsive microenvironment for growing tissues. Our above comprehensive results may be widely expanded to other areas of tissue engineering in wound healing and oxidative stress related skin protection [10].

## Conclusions

In summary, we present a simple route to developing a chemical combination of AST with GO sheets to reinforce structural stability and antioxidant activity in biological environments. As a result, the bioabsorbable multifunctional ultrathin nanostructured film was produced by the self-assembly process of 2D nanomaterials constructed with a specified chemical conformation. The highly stable physicochemical properties of AST in this design were obviously sustainable due to their amphipathic structure, which accesses various biomolecules through noncovalent interactions. AST has a strong antioxidant effect because of its radical-trapping double-bonded polyene chain, and this unique chemical structure showed an excellent ability to scavenge ROS, which can regulate the redox balance in wound healing. Since ROS plays an important role in wound healing and is involved in all steps, the balance and regulation between the generation and elimination of ROS are essential and critical [72]. GO has a significant antioxidant effect in the form of radical scavenging, which can protect various biomolecules from oxidation. Cotreatment of GO-AST solution demonstrated high ABTS and DPPH radicals scavenging activity and a high antioxidant combined effect by AST molecules tightly bound to the GO sheets as a biocompatible nanocarrier. As another approach, the GO/RGD/AST structured film also exhibited excellent antibacterial effects based on physical damage against *E. coli* and *S. aureus*. Therefore, the newly developed wound healing dressing material could be effective with functions such as antioxidant, antibacterial, and anti-inflammatory. In a reduced intracellular oxidative stress, cell functions can be maintained by the promotion of fibroblast migration and proliferation, enhancing the wound healing process. Additionally, the viability was restored by suppressing the toxicity of LPS-induced cells, which can restrict cell apoptosis mediated by inflammatory and promoting migration of the main component cell in the skin. With these summarized results, The AST can overcome the low bioavailability by combining with GO and RGD peptide, and maximizing their respective advantages as a nanocarrier, based on stronger antioxidant, antibacterial, and anti-inflammatory effects. It can participate in cell proliferation and inflammatory processes to help wound healing and can be used very effectively; therefore, can apply a new nanocarrier to the therapy for skin diseases including wound healing [73, 74]. Furthermore, it can treat various inflammatory diseases mediated by oxidative stress; therefore, it is potential for various applications in the field of tissue engineering.

## Supplementary Information

**Additional file 1: Figure S1.** The basic chemical structure of AST composed of non-polar polyene and two polar end groups; their main reactions were induced by the ROS, such as singlet oxygen (^1^O_2_), and hydroxyl (OH^•^), and superoxide (O_2_^•-^) radicals. **Figure S2.** The fabrication process of GO-based multifunctional patch; GO/RGD/AST film on glass substrate was transferred to HA film. **Figure S3.** The tensile strength measurement on GO/RGD/AST-based wound dressing patch; the inset images show morphological changes of samples at stress-strain curve. **Figure S4.** TEM images and histograms of the particle size distribution for (a) RGD peptide- GO nanosheets and (b) AST-GO nanosheets. **Figure S5.** A collective set of results from the use of Raman spectroscopy separately on the GO, GO/RGD, GO/AST, and GO/RGD/AST films in the range of 800-2400 cm^-1^. **Figure S6.** Cell area change of H_2_O_2_-induced L-929 cell on the GO, GO/RGD, GO/AST, and GO/RGD/AST films. **Figure S7.** The generation routes of ROS by energy transfer; metal-catalyzed reaction such as Fenton reaction and Haber-Weiss reaction convert hydrogen peroxide (H_2_O_2_) and superoxide (O_2_^•-^) into hydroxyl (OH^•^) radical and peroxyl radical (ROO^•^).

## Data Availability

All data generated or analyzed during this study are included in this published article.

## Abbreviations

AST: Astaxanthin
GO: Graphene oxide
UV: Ultraviolet
ROS: Reactive oxygen species
2D: Two-dimensional
FESA: Flow-enabled self-assembly
ATP: Adenosine triphosphate
HA: Hyaluronic acid
APTES: 3-Aminopropyltriethoxysilane
DI: Deionized
DMSO: Dimethyl sulfoxide
EDC: N-(3-dimethylaminopropyl)-N′-ethylcarbodiimide
NHS: N-hydroxysuccinimide
FT-IR: Fourier-transform infrared spectroscopy
ATR: Attenuated total reflectance
TEM: Transmission electron microscopy
SEM: Scanning electron microscopy
AFM: Atomic force microscopy
RMS: Root mean square
PBS: Phosphate buffered saline
H_2_O_2_: Hydrogen peroxide
ABTS: 2,2′-Azino-bis(3-ethylbenzothiazoline-6-sulfonic acid
DPPH: 2.2-Diphenyl-1-picrylhydrazyl
FBS: Fetal bovine serum
LPS: Lipopolysaccharide
NO: Nitric oxide
