# Supplementary material for: Surface-mediated high antioxidant and anti-inflammatory effects of astaxanthin-loaded ultrathin graphene oxide film that inhibits the overproduction of intracellular reactive oxygen species
Source: Biomater Res. 2022 Jul 6;26:30. doi: 10.1186/s40824-022-00276-4 (PMC9258176; doi:10.1186/s40824-022-00276-4)
Supplement: Supplementary file 1 — Additional file 1: Figure S1. The basic chemical structure of AST composed of non-polar polyene and two polar end groups; their main reactions were induced by the ROS, such as singlet oxygen (1O2), and hydroxyl (OH•), and superoxide (O2•-) radicals. Figure S2. The fabrication process of GO-based multifunctional patch; GO/RGD/AST film on glass substrate was transferred to HA film. Figure S3. The tensile strength measurement on GO/RGD/AST-based wound dressing patch; the inset images show morphological changes of samples at stress-strain curve. Figure S4. TEM images and histograms of the particle size distribution for (a) RGD peptide- GO nanosheets and (b) AST-GO nanosheets. Figure S5. A collective set of results from the use of Raman spectroscopy separately on the GO, GO/RGD, GO/AST, and GO/RGD/AST films in the range of 800-2400 cm-1. Figure S6. Cell area change of H2O2-induced L-929 cell on the GO, GO/RGD, GO/AST, and GO/RGD/AST films. Figure S7. The generation routes of ROS by energy transfer; metal-catalyzed reaction such as Fenton reaction and Haber-Weiss reaction convert hydrogen peroxide (H2O2) and superoxide (O2•-) into hydroxyl (OH•) radical and peroxyl radical (ROO•). [file 40824_2022_276_MOESM1_ESM.docx]

**Supporting information**

Surface-mediated high antioxidant and anti-inflammatory effects of astaxanthin-loaded ultrathin graphene oxide film that inhibits the overproduction of intracellular reactive oxygen species

*Seon Yeong Chae*,^a^ *Rowoon Park*,^b^ and *Suck Won Hong* ^b,^*^*^*

**^a^** Engineering Research Center for Color Modulation Extrasensory Cognitive Technology, Pusan National University, Busan 46241, Republic of Korea

**^b^** Department of Cogno-Mechatronics Engineering, Department of Optics and Mechatronics Engineering, College of Nanoscience and Nanotechnology, Pusan National University, Busan 46241, Republic of Korea

*Correspondence should be addressed to S.W.H. (email: swhong@pusan.ac.kr)


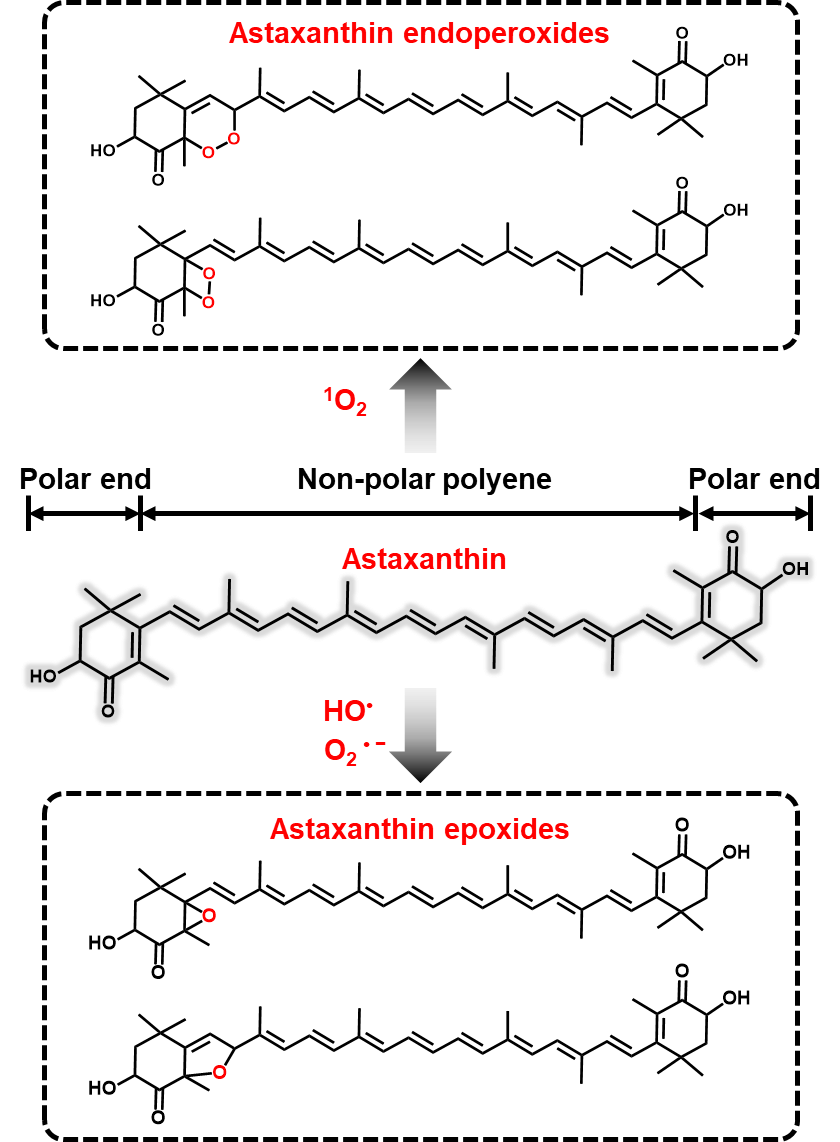


**Fig. S1.** The basic chemical structure of AST composed of non-polar polyene and two polar end groups; their main reactions were induced by the ROS, such as singlet oxygen (^1^O_2_), and hydroxyl (OH^•^), and superoxide (O_2_^•-^) radicals.


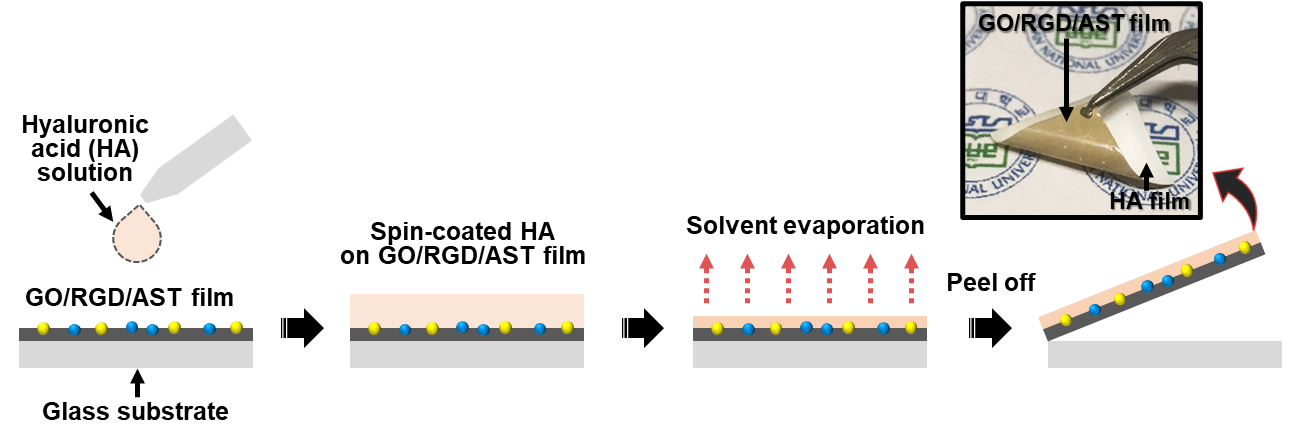
**Fig. S2.** The fabrication process of GO-based multifunctional patch; GO/RGD/AST film on glass substrate was transferred to HA film.


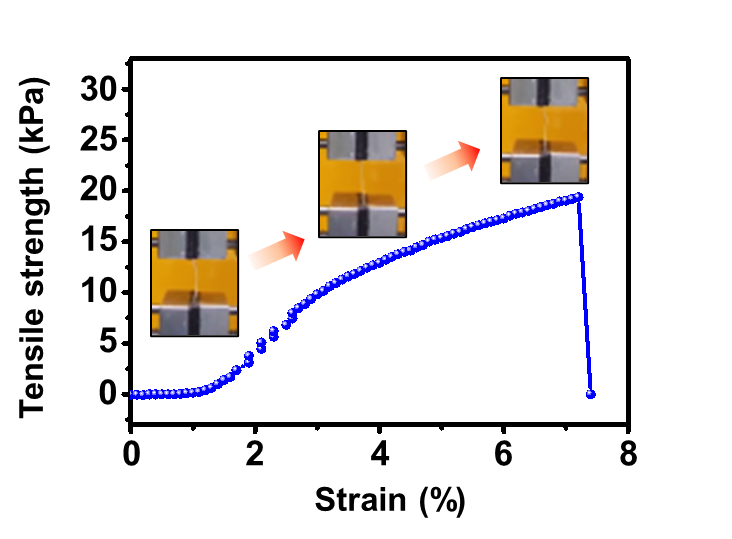


**Fig. S3.** The tensile strength measurement on GO/RGD/AST-based wound dressing patch; the inset images show morphological changes of samples at stress-strain curve.


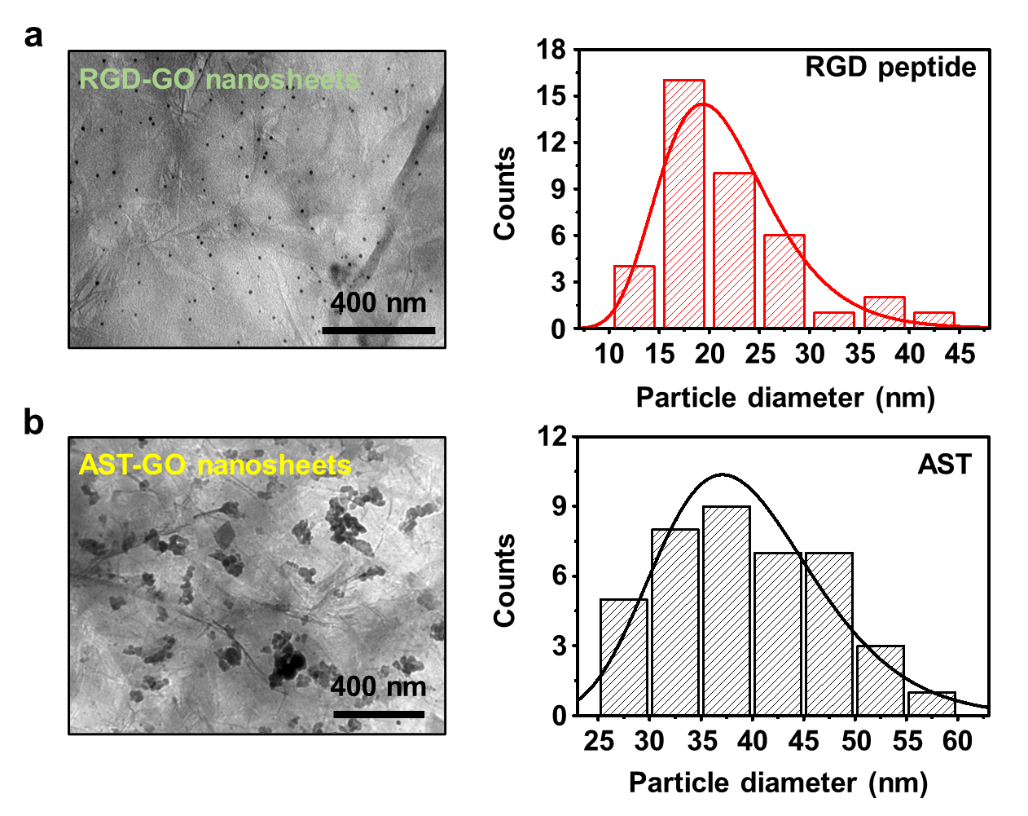


**Fig. S4.** TEM images and histograms of the particle size distribution for (a) RGD peptide- GO nanosheets and (b) AST-GO nanosheets.


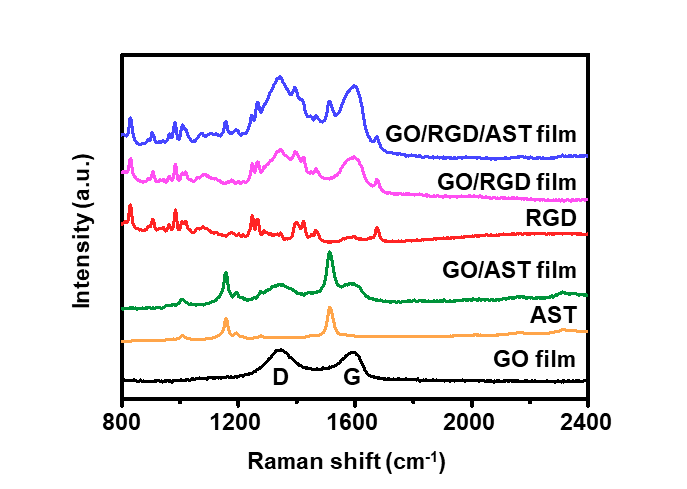


**Fig. S5.** A collective set of results from the use of Raman spectroscopy separately on the GO, GO/RGD, GO/AST, and GO/RGD/AST films in the range of 800-2400 cm^-1^.

**Fig. S6.** Cell area change of H_2_O_2_-induced L-929 cell on the GO, GO/RGD, GO/AST, and GO/RGD/AST films.


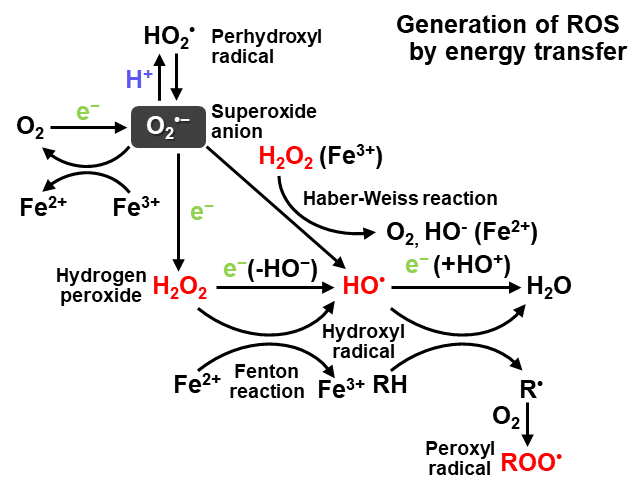


**Fig. S7.** The generation routes of ROS by energy transfer; metal-catalyzed reaction such as Fenton reaction and Haber-Weiss reaction convert hydrogen peroxide (H_2_O_2_) and superoxide (O_2_^•-^) into hydroxyl (OH^•^) radical and peroxyl radical (ROO^•^).
